# The Investigation of Thymol Formulations Containing Poloxamer 407 and Hydroxypropyl Methylcellulose to Inhibit *Candida* Biofilm Formation and Demonstrate Improved Bio-Compatibility

**DOI:** 10.3390/ph15010071

**Published:** 2022-01-05

**Authors:** Enas Al-Ani, Wayne Heaselgrave

**Affiliations:** 1 Research Institute in Healthcare Science, Faculty of Science and Engineering, University of Wolverhampton, Wolverhampton WV1 1LY, UK; 2 Department of Biomedical Science, University of Wolverhampton, Wolverhampton WV1 1LY, UK

**Keywords:** thymol, hydroxypropyl methyl cellulose, poloxamer 407, *Candida albicans*, biofilm inhibition, antibiofilm, biocompatibility

## Abstract

The aim of this study was to investigate the potential of thymol to inhibit *Candida* biofilm formation and improve thymol biocompatibility in the presence of hydroxypropyl methylcellulose (HPMC) and poloxamer 407 (P407), as possible drug carriers. Thymol with and without polymers were tested for its ability to inhibit biofilm formation, its effect on the viability of biofilm and biocompatibility studies were performed on HEK 293 (human embryonic kidney) cells. Thymol showed a concentration dependent biofilm inhibition; this effect was slightly improved when it was combined with HPMC. The Thymol-P407 combination completely inhibited the formation of biofilm and the antibiofilm effect of thymol decreased as the maturation of *Candida* biofilms increased. The effect of thymol on HEK 293 cells was a loss of nearly 100% in their viability at a concentration of 250 mg/L. However, in the presence of P407, the viability was 25% and 85% using neutral red uptake and sulforhodamine B assays, respectively. While, HPMC had less effect on thymol activity the thymol-P407 combination showed a superior inhibitory effect on biofilm formation and better biocompatibility with human cell lines. The combination demonstrates a potential medical use for the prevention of *Candida* biofilm formation.

## 1. Introduction

Thymol is a phenolic compound present in the essential oil extracted from the culinary herb Thyme (Thymus vulgaris). It is a member of the Lamiaceae family and is one of the most common ingredients found in antiseptic mouthwashes [1]. Its antifungal mechanism of action occurs through its ability to increase the fluidity and permeability of membrane lipids which interferes with hyphael production [2]. The major reason for pathogenicity in *C. albicans* is attributed to its ability to form a biofilm, which is more resistant to antifungals [3]. In previous work, it was found that thymol significantly decreased the adhesion of *C. albicans* to epithelial cells and such adhesion is considered the first step in pathogenesis [4].

The biocompatibility of thymol has been reported by various researchers including the ability to induce chromatin condensation and DNA cleavage in human gastric carcinoma cells (AGS), leading to depolarisation of mitochondrial membrane potentials and apoptosis [5]. Moreover, in Caco-2 cells, thymol caused lipid degeneration, mitochondrial damage, nucleolar segregation and apoptosis [6].

The inhibition of biofilm formation is paramount to prevent chronic infection. Different studies have investigated the effectiveness of polymer-antimicrobial combinations to inhibit biofilm formation. In a previous study, polymethylmethacrylate was observed to improve the activity of nystatin, polymyxin B and chlorhexidine to inhibit *C. albicans* biofilm formation [7]. However, no improvement was found in the antimicrobial activity of gentamycin and vancomycin when combined with poloxamer against *Staphylococcus aureus* and *S. epidermidis*, although, the polymer inhibited the adhesion of both microorganisms [8]. In another study, poloxamer 338 showed a 100% inhibition of *Escherichia coli* biofilm formation on silicone urinary catheters [9].

In the current study, the impact of hydroxypropyl methylcellulose (HPMC) and poloxamer 407 (P407) on the effect of thymol was investigated. HPMC has been included in commercially available products and is thoroughly investigated in buccal drug delivery [10]. Furthermore, P407 is widely used in several pharmaceutical preparations and it is one of the ingredients in Listerine^®^ mouthwash (Johnson & Johnson, Louis, MO, USA) [11]. In this present study, the antifungal activity and the biocompatibility of thymol were investigated in an attempt to evaluate the feasibility of formulating thymol for controlled buccal delivery in the treatment of oral candidiasis. The investigations were undertaken for two hours to evaluate both the efficacy and the safety of thymol in the presence of the carriers HPMC and P407.

## 2. Results

### 2.1. Minimum Inhibitory Concentration (MIC) and Minimum Biocidal Concentration (MBC)

Neither HPMC nor P407 showed antifungal activity at a concentration range of 0.005 to 5000 mg/L. The MIC and MBC of thymol were 125 and 250 mg/L, respectively. Thymol-P407 at 1:5 ratio increased the MBC of thymol above 250 mg/L with no effect on the MIC. Thymol-P407 at ratios 1:1 to 1:4 have the same MIC and MBC of thymol. However, no observed effect of HPMC on the activity of thymol

### 2.2. Effect on the Inhibition of Biofilm Formation

**Figure 1:**
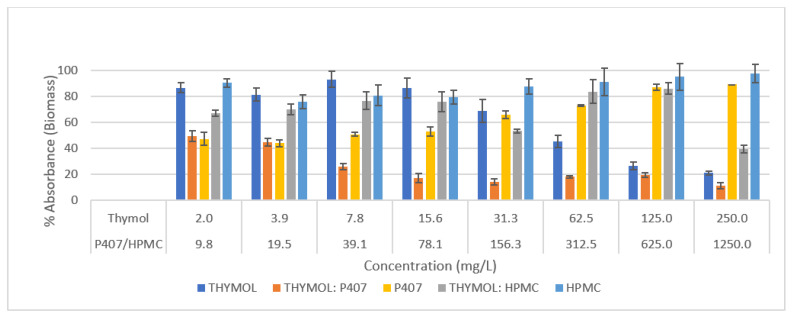
Effect of thymol, Thymol-P407, P407, Thymol-HPMC and HPMC on the inhibition of *C. albicans* biofilm formation (*n* = 4) for 4 h at 37 °C using Crystal Violet assay. Each point represents the mean percentage ± SE.

**Figure 2:**
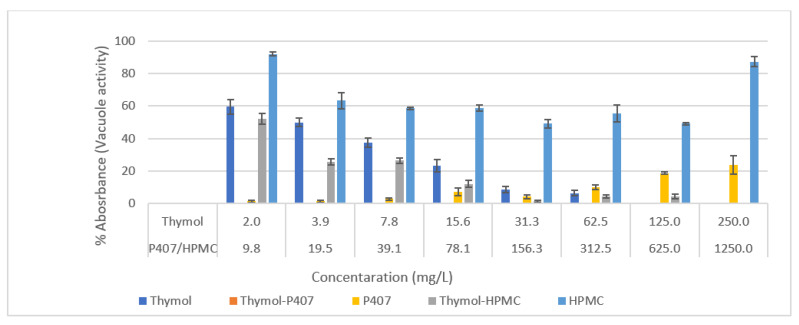
Effect of thymol, Thymol-P407, P407, Thymol-HPMC and HPMC on the inhibition of *C. albicans* biofilm formation (*n* = 4) for 4 h at 37 °C using Neutral red uptake assay. Each point represents the mean percentage ± SE.

Based on the MIC and MBC results; thymol, Thymol-P407 and Thymol-HPMC at ratios of 1:5 were investigated for the rest of the analyses. P407 and HPMC at five times the concentration of thymol were tested for their inhibitory effect on *Candida* biofilm. In the crystal violet assay (CV), the %absorbance of thymol: P407 was less than 20% at a concentration of ≥15.6 mg/L with no viability detected using neutral red uptake assay (NR) at the investigated concentrations (Figure 1 and Figure 2). The P407 inhibitory effect was less compared to Thymol-P407 and the effect decreased with increasing P407 concentration. The thymol:P407 has inhibited the biofilm formation at thymol concentration range of 2–250 mg/L (poloxamer 10–1250 mg/L). These concentrations are above and below the critical micelle concentration (CMC) of P407 which means the CMC has no impact on the activity against *C.albicans* (Figure 1 and Figure 2). HPMC-thymol combination showed high biomass which might be attributed to the high viscosity and mucoadhesive property of HPMC. HPMC-thymol showed around a 50% reduction in viability at a concentration of ≥3.9 mg/L compared to thymol. Moreover, thymol had a concentration dependent biofilm inhibitory formation effect. *C. albicans* biofilm formed in the presence of HPMC maintained around 60% of its viability based on NR assay and around 80% of its biomass using CV assay at most of the investigated concentrations (Figure 1 and Figure 2).

### 2.3. Effect of Thymol on C. albicans Biofilm

**Figure 3:**
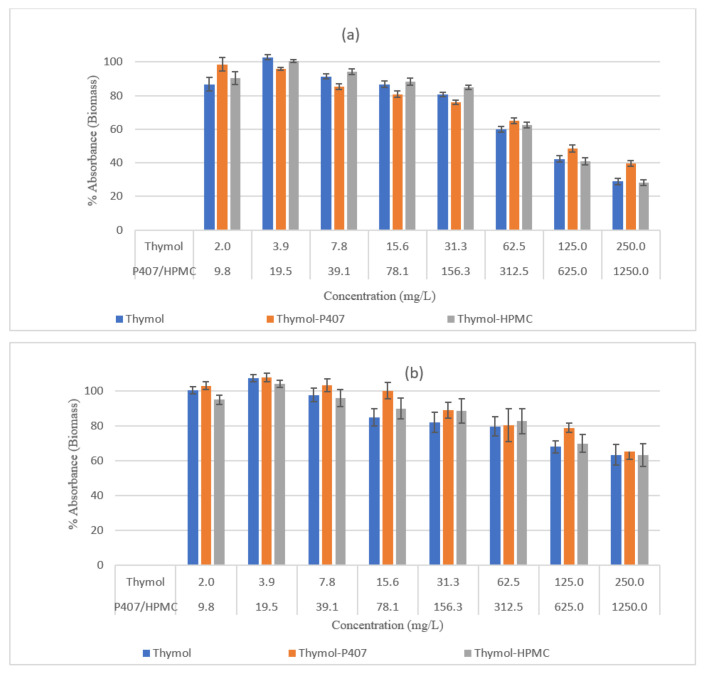
Effect of thymol on the biomass of *C. albicans* biofilm (**a**) 4 h, (**b**) 24 h, (*n* = 8) for 2 h at 37 °C. Each point represents the mean percentage ± SE.

In the crystal violet (CV) assay, biomass loss increased with increasing thymol concentration. The effect of the MBC and MIC on the 4-h biofilm, is shown in Figure 3a. The biomass percentages were 29% and 42%, respectively, and at 62.5 mg/L it increased to 60%. As thymol concentrations decreased, there was a gradual increase in the biomass with no effect was registered at 3.91 mg/L. However, the 24-h biofilm was more resistant with 63% and 68% of the biofilm remaining at planktonic MBC and MIC values respectively (Figure 3b). Moreover, at concentrations of ≥7.81 mg/L, 100% of the biomass was retained, which showed that it was more resistant than the 4-h biofilm. This indicates that thymol is more affective against early biofilm formation.

**Figure 4:**
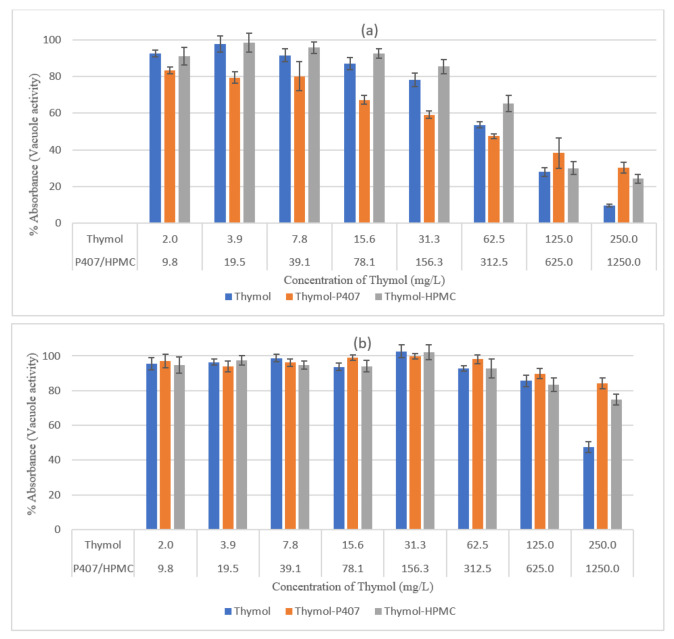
Effect of thymol on *C. albicans* biofilms using neutral red uptake assay (**a**) 4 h, (**b**) 24 h, (*n* = 6) for 2 h at 37 °C. Each point represents the mean percentage ± SE.

The effect of thymol on the activity of vacuoles (viability) is presented in Figure 4. In *C. albicans* biofilm at 4 h it was observed that it decreased the apparent viability at concentrations greater than 15.63 mg/L. For instance, at 125 and 250 mg/L, the viability was 28% and 10%, respectively. The P407 antagonised the effect of thymol at 125 and 250 mg/L. The antagonistic effect of P407 at 250 mg/L (planktonic MBC) was equivalent to that on planktonic cells as it increased the MBC to >250 mg/L. On the other hand, P407 showed an increase in thymol activity at a concentration of ≤62.5 mg/L. HPMC showed a similar antagonistic effect only at 250 mg/L.

**Figure 5:**
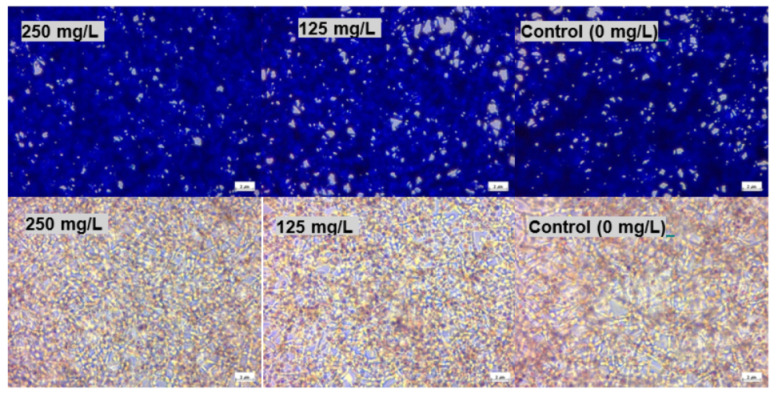
Images of C. albicans biofilm 24-h treated with thymol and stained with crystal violet assay (**top** row) and neutral red assay (**bottom** row) (×400).

In Figure 4b, *C. albicans* biofilm at 24 h was more resistant to thymol compared to the 4-h biofilm, in which loss of cell viability occurred only at concentrations ≥ 125 mg/L. Both polymers decreased the antimicrobial activity of thymol at 250 mg/L. There was no distinctive difference between the light microscopy micrographs of the biofilm at 24 h and the control when stained with NR and CV, even at a higher concentration of thymol (250 mg/L) (Figure 5).

### 2.4. Biocompatibility Assay

**Figure 6:**
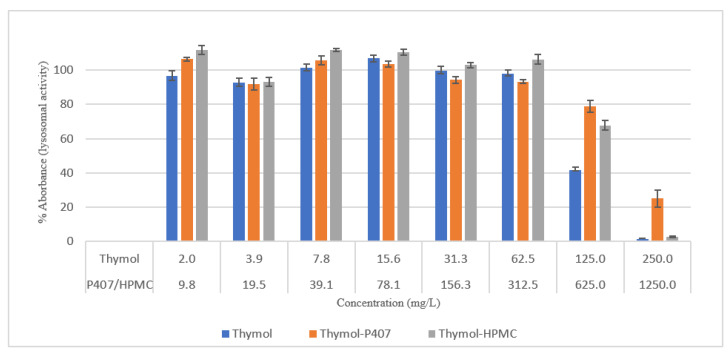
Effect of thymol on the lysosomal activity of HEK293 cells, 2-h exposure, measured using neutral red uptake assay. Each point represents the mean percentage ± SE, *n* = 6.

The effect of thymol with and without HPMC or P407 was investigated using HEK293 cells (human embryonic kidney cells). A 100% loss in the Lysosomal activity was shown by thymol and Thymol-HPMC at 250 mg/L compared to 25% activity with Thymol-P407. Furthermore, at a concentration of 125 mg/L, the combination of thymol with either polymer nearly doubled the activity of lysosomes compared to thymol alone (Figure 6). 

**Figure 7:**
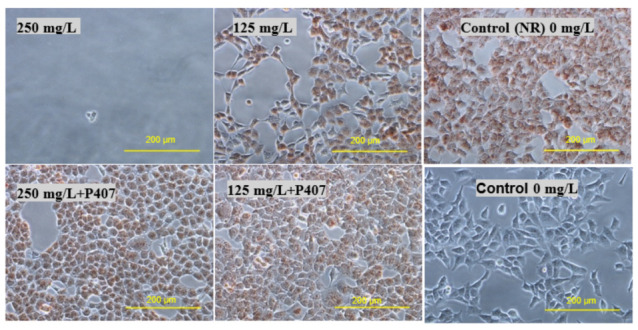
Micrographs of HEK293 cells treated with thymol and Thymol-P407 at a concentration of 125 and 250 mg/Land stained and with NR (×200).

**Figure 8:**
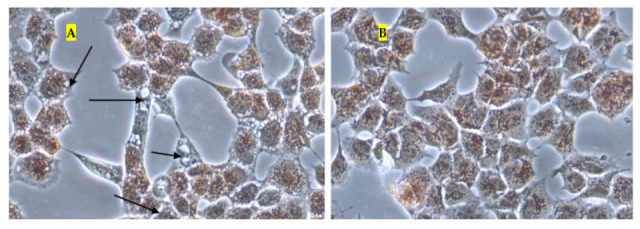
Micrographs of HEK293 (**A**) cells treated with Thymol-P407 250 mg/L (**B**) untreated cells (control) and stained with neutral red uptake assay (×200) (arrows showing vacuolization).

The effect of thymol and Thymol-P407 on HEK293 cells when treated with 125 and 250 mg/L are depicted in the micrographs (Figure 7). No significant difference is noticed at a concentration of 125 mg/L between the images of thymol and thymol-P407. However, cells treated with 250 mg/L thymol only, resulted in their detachment and no cells are shown in the image after staining with NR (Figure 7). However, when the Thymol-P407 cells are visualised, they showed vacuolization of the cytoplasm compared to the control (Figure 8).

**Figure 9:**
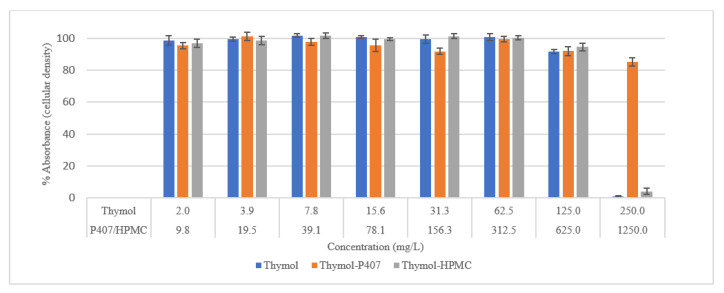
Effect of thymol on the viability of HEK293 cells, 2-h exposure, measured using a Sulforhodamine B assay. Each point represents the mean percentage ± SE, *n* = 9.

Using the Sulforhodamine B assay, in Figure 9, the effect of thymol at 250 mg/L on the cells was observed. The thymol-P407 retained 85% of the cellular density compared to less than 3% with the thymol-HPMC, the P407 protected the cells from the effect of thymol. This result agreed with the NR assay results. 

**Figure 10:**
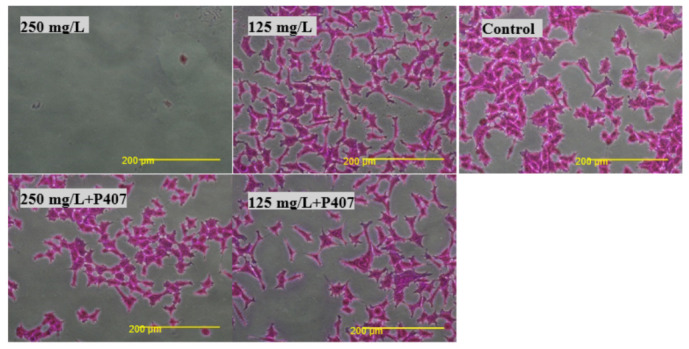
Micrographs of HEK293 cells treated with Thymol and Thymol-P407 at a concentration of 125 and 250 mg/L for two hours at 37 °C and stained with SRB (×200).

The micrographs of the HEK293 cells treated with thymol and thymol-P407 at 125 and 250 mg/L are displayed in Figure 10. No morphological variation was observed between the different treatments except the detachment of the cells when treated with 250 mg/L of thymol. 

## 3. Discussion

The antifungal effects of thymol on *C. albicans* planktonic and biofilm cells are equally important. Although the pathogenicity of *C. albicans* is primarily attributed to biofilm formation, the first stage of its pathogenesis starts with the adhesion of yeast cells to surfaces including the buccal mucosa [3]. For planktonic cells, the MBC was 250 mg/L on *C. albicans* (ATCC 10231, Manassas, VA, USA); whereas the MIC was 125 mg/L and it is identical with that found in a previous study when the effect of thymol was instigated on *C. albicans* [2]. Although the used polymers are bioadhesive, the HPMC did not impact the MBC of thymol, while thymol-P407 increased it 250 mg/L to ≥250 mg/L which may be explained by its surfactant activity [11]. The P407 increased the solubility of thymol in the aqueous medium and interfered with its ability to be incorporated into the cell membrane and disrupt the phospholipid bilayer [12].

In a recent study looking at the effect of flavours on gelling temperature and the viscosity of P407, the authors found an interaction between thymol and P407; the former changed the thermal behaviour and the gelling temperature of the latter [13]. In another study, the authors found that the non-ionic surfactant Tween 80 counteracted the effect of thymol against the bacterium *Salmonella typhimurium* and the inhibitory effect increased with an increasing concentration of Tween 80. The authors hypothesised that the solubility of thymol in an aqueous media was increased upon the addition of the surfactant, leading to a decrease in thymol solubility in the *S. typhimurium* cell membrane [14]. 

In the current study, the effect of thymol on *Candida* biofilm was analysed using CV and NR assays. CV measure the biomass of both dead and live cells in biofilms. Consequently, it does not reflect the degree of inactivation of the cells in the biofilm, but its mass left after treatment [15]. NR reflected the viability of the cells; due to its basic properties, it is accumulated in the viable vacuoles of fungi or lysosomes of cells. Due to their low pH compared to the cytoplasm, NR retention in these organelles stains them red [16]. This assay is widely used to measure the biocompatibility of human cell lines, and it was used in this work for testing *Candida* biofilms since the pH of the vacuoles is around 5.5 [17]. The pH gradient is maintained in healthy living cells; however, the pH gradient will be lost when the cell dies and subsequently lysosomes and vacuoles are not able to retain the dye [16].

The P407 and HPMC both improved the biofilm inhibitory effect of thymol and the thymol-P407 combination showed a 100% biofilm inhibitory formation at all investigated concentrations based on NR assay. The effect of P407 and thymol is additive, the biofilm inhibitory activity of poloxamer was recorded at the lower concentrations and thymol at the higher concentrations. The inhibitory effect of poloxamer is inversely proportional to its concentration, which might be attributed to the self-association of poloxamer resulting in decreasing the interaction of the polymer with the *Candida* (Figure 1 and Figure 2) [18]. P407 is a nonionic surfactant polymer and is known to inhibit bacterial aggregation by suppressing protein aggregation in the biofilm matrix. Thymol-HPMC and Thymol showed 25 and 50% biofilm formation at 3.9 mg/L (Figure 1). The effect of HPMC on biofilm inhibitory formation is attributed to its properties as a suspending and thickening agent. Thymol interferes with the production of hyphae and it showed an inhibitory effect of 80% on *C. albicans* biofilm development after treatment with 600 mg/L of thymol for 24 h [2,19].

The effect of Thymol on the vacuolar activity and biofilm mass was concentration dependent, and its effectiveness decreased with an increase in the maturation of the biofilm. The effect on the biomass was maximal with 250 mg/L, leaving the biofilm with 29% and 63% biomass for 4-h and 24-h biofilms, respectively (Figure 3), the decrease in thymol concentrations led to a decrease in the reduction of biomass. Moreover, at a concentration of 250 mg/L the vacuolar activity was 10% and 47% for the 4-h and 24-h biofilms, respectively (Figure 4). It was found that thymol interfered with hyphael production by interacting with *C. albicans* and as a consequence, it changed the cell membrane morphology leading to an increase in fluidity and permeability [20]

The activity of thymol was decreased when combined with HPMC at 250 mg/L and tested using neutral red assay, at lower concentrations this effect was diminished (Figure 4). This effect was not observed on planktonic cells and when using CV analysis and the inconsistency of the results might be attributed to the mucoadhesive nature of HPMC. The P407 had no impact on the activity of thymol when tested by CV assay, which might be attributed to the measurement of the mass of both dead and living cells. However, when assessed by NR assay, thymol activity decreased at 250 mg/L of thymol for the 4-h and 24-h biofilms, a result similar to that for planktonic cells. Accordingly, HPMC had no effect on the activity of thymol whereas high concentrations of P407 decreased thymol activity.

Thymol is considered cytotoxic to HEK293 cells at 125 and 250 mg/L [20], and it was highly antagonised by P407 (Figure 6). This observation is supported by a previous investigation which showed no effect on the mitochondrial activity of neuronal cells when treated with thymol at a concentration of 150 mg/L for 30 min and 24 h using an MTT assay [21]. However, there was a 40% reduction in the viability of human blood cells at concentrations of 150 and 200 mg/L and tested using MTT assay [22].

In this study, the viability of HEK293 cells treated with thymol and investigated using NR showed a suppression of activity of nearly 100% at 250 mg/L with approximately 40% of activity remaining at 125 mg/L. P407 presented similar protective pattern with lower viability (Figure 6). This observation agrees with a previous study, which found that the toxicity of tramadol has decreased approximately two-fold when combined with P407 and tested using MTT and NR uptake assays [23]. In Figure 7 and Figure 10, the micrographs of cells treated with 250 mg/L of thymol show the disappearance of the cells due to their detachment by the effect of thymol, which might be explained by anoikis, which is a type of cell death induced upon their detachment from the neighbouring cells and extracellular matrix [24].

Whereas when thymol at the same concentration combined with P407 resulted in the protection of the cells from being detached. Previously poloxamers have been found to promote wound healing by sealing the pores of the cellular membrane helping to maintain cellular integrity [25]. Although, P407 protects the cells from detaching it did not inhibit their vacuolization (Figure 8). The opposing effect of P407 to thymol on biofilm might be attributed to the same mechanism of healing in a human cell line. In this study cytoplasmic vacuolization confirms a previous investigation performed on Caco-2 cells which were treated with thymol for 48 h at a concentration of 37.5 mg/L. Cell detachment was observed with carvacrol but not with thymol in the same investigation, which might be due to the lower concentration of thymol used in that study (37.5 mg/L). Both thymol and carvacrol have very similar chemical structures, and both are constituents in thyme oil. 

Furthermore, in a previous study, the effect of thymol on Caco-2 cells was investigated. The authors observed cellular necrosis when they were treated with 120 mg/L at approximately 10 and 15% after 1 and 24 h, respectively, with apoptosis of less than 1% [26]. However, in the current investigation, cell death is attributed to apoptosis as a consequence of anoikis and vacuolization with no reported sign of necrosis. This toxic effect is concentration-dependent, and it was found after two hours treatment with 250 mg/L of thymol.

Biocompatibility was also tested using an SRB assay. SRB is used to measure the density of cells based on the cellular protein content of dead and live cells [27]. Cells treated with thymol and thymol-HPMC showed a substantial loss of proteins at a concentration of 250 mg/L, and this is due to cells detachment. Thymol-P407 showed a potent biofilm inhibitory formation at a low concentration of 2 mg/L. This effect is considered a promising effect in the prophylaxis treatment of oral candidiasis by inhibiting biofilm formation. 

## 4. Materials and Methods

### 4.1. Test Compounds

Thymol (Alfa Aesar, Heysham, UK), Poloxamer 407 (P407) (Sigma-Aldrich, Gillingham, UK) and Methocel^TM^ F4M (HPMC, which was a kind gift from Colorcon, Dartford, UK). Thymol was serially diluted from 250 to 1.95 mg/L and Thymol-HPMC and Thymol-P407 were co-dissolved individually with thymol at a ratio of 5:1. 

### 4.2. C. albicans Strain and Inoculum Preparation

*C. albicans* (ATCC 10321, Virginia Manassas, VA, USA) were grown on Sabauroud dextrose agar (SDA, Sigma-Aldrich, Gillingham, UK) and incubated at 30 °C for 18–24 h. Then, few colonies were transferred to Muller Hinton broth (MHB, Sigma-Aldrich, Gillingham, UK). The optical density was equivalent to 0.5 McFarland standard solution, and the concentration of the *C. albicans* overnight culture was 1–5 × 10^6^ CFU/mL. 

### 4.3. Minimum Inhibitory Concentration (MIC) and Minimum Biocidal Concentration (MBC)

The broth macro dilution method was used to test the MIC. A two-fold dilution of thymol with and without P407 and HPMC was performed in MHB to a final volume of 5 mL. Then, each tube was inoculated with 200 µL of the inoculum (giving a final cell density of 1 × 10^4^–10^5^ CFU/mL) then incubated at 30 °C for 24–48 h. 

The MBC was tested on SDA plates by inoculating 10 µL of broth from the tubes which showed no visible growth (MIC). The plates were incubated at 30 °C for 24–48 h.

### 4.4. C. albicans Biofilm Formation

The pH of the RPMI-1640 medium (Sigma-Aldrich, Gillingham, UK) (RPMI) was adjusted to 7.2 with 0.165 M MOPS (3-morpholinopropane-1-sulfonic acid (Sigma-Aldrich, Gillingham, UK). RPMI-1640 medium was seeded with *C. albicans* to a final concentration of 5×10^5^ CFU/mL. The 96-well microtiter plate was inoculated with 200 µL/well and incubated at 37 °C for 4 h for initial biofilm formation and 24 h for a mature biofilm) [28]. 

### 4.5. Inhibition of Biofilm Formation

Thymol with and without HPMC was serially double diluted in RPMI-1640 in a 96-well plate at a concentration range of 2–250 mg/L. Then *Candida* suspension was added to the wells and incubated at 37 °C for four hours. The inhibition was tested using NR and CV assays as explained below.

### 4.6. Thymol Antibiofilm Activity

The biofilms were washed twice with 200 µL of phosphate buffer saline (PBS) (10 mM, pH = 7.4) to remove non-adherent cells. The washed biofilms were cultured for two hours at 37 °C with pre-prepared serial double dilutions of the tested compounds. Which, then removed and the treated biofilm was washed with 200 µL of PBS and the antibiofilm activity was determined using neutral red uptake and crystal violet assays.

### 4.7. Neutral Red Uptake (NR) Assay (C. albicans Biofilm)

Neutral red (NR, 3-amino-7-dimethylamino-2-methyl-phenazine hydrochloride, Sigma-Aldrich, Gillingham, UK) (NR) assay has been described in detail in earlier studies [16] Briefly, after washing biofilm with PBS, aliquots of 100 µL of NR (80 mg/L in PBS) was added to each well to determine the vacuole activity, and the plate was incubated at 37 °C for two hours. The stain was removed, and the biofilms were washed with 150 µL of PBS. Then was fixed for 2 min with 100 µL of 5% glutaraldehyde (Alfa Aeser, Heysham, UK). NR was extracted from the vacuoles with 150 µL of de-stain solution (50% absolute ethanol, 48% ultrapure water and 2% glacial acetic acid). Then the plate was placed on an orbital shaker for 30 min to extract the NR from the biofilm. Finally, a 100 µL aliquot was transferred from each well into a new microtiter plate and the absorbance was measured at λ_540nm_ (Multiskan Ascent, Thermo Labsystems, Vantaa, Finland). Biofilm viability was calculated as a percentage of untreated cells. 

### 4.8. Crystal Violet (CV) Assay

Absolute ethanol was used to fix the washed biofilms, with a volume of 100 µL/well was added and left for 15 min, then the latter was removed, and the microtiter plate was left to air-dry. The CV stain (Chadwell Heath, London, UK) was prepared at a concentration of 0.1% (*w*/*v*) and aliquots of 100 µL were added to the wells for 20 min. Then the CV was removed, and the biofilms were washed under a gentle stream of running tap water. Acetic acid (Sigma-Aldrich, Gillingham, UK) was used to extract the CV from the biofilm by adding 150 µL/well. Then, from each well a 100 µL of the supernatant was moved to a new plate, and the absorbance was recorded at λ_595nm_ using a plate reader (BioTec, EL800, Dorset, UK). The biomass was calculated as a percentage of untreated cells.

### 4.9. Cell Line and Culture Medium

The in vitro biocompatibility was tested on HEK293 cells (Human embryonic kidney cells). Dulbecco’s Modified Eagle’s Medium (DMEM) was supplemented with 10% (*v*/*v*) Foetal Bovine Serum (FBS), 1% (*v*/*v*) L-Glutamine solution (200 mM) and 1% (*v*/*v*) Antibiotic-Antimycotic Solution (10,000 U penicillin, 10 mg streptomycin and 25 μg amphotericin B per mL) (Sigma-Aldrich, Gillingham, UK).

### 4.10. Biocompatibility Assays

The biocompatibility assay was performed by inoculating a 96-well microtiter plate with HEK293 cells (4.0 × 10^4^/well). The plate was incubated overnight at 37 °C in a humidified incubator with 5% CO_2_. Then DMEM was replaced with the test compounds, which were previously two-fold diluted in DMEM and the plate was incubated for two hours. The cell viability was evaluated using NR and Sulforhodamine B (SRB) assays.

### 4.11. Neutral Red Uptake (NR) Assay (HEK 293 Cells)

The NR was prepared in DMEM (40 mg/L) then aliquots of 100 µL of NR were added to each well of pretreated HEK 293 cells as explained in Section 4.10 to determine the lysosomal activity, and the plate was incubated at 37 °C for two hours. The stain was removed, and the cells were washed with 150 µL of PBS. This was then fixed for 2 min with 100 µL of 5% glutaraldehyde. NR was extracted from the lysosomes with 150 µL of de-stain solution (50% absolute ethanol, 49% ultrapure water and 1% glacial acetic acid). Then the plate was placed on an orbital shaker for 30 min to extract the NR from the biofilm. Finally, a 100 µL aliquot was transferred from each well into a new microtiter plate and the absorbance was measured at λ_540nm_ (Multiskan Ascent, Thermo Labsystems, Finland). Biofilm viability was calculated as a percentage of untreated cells. 

### 4.12. Sulforhodamine B (SRB) Assay

SRB assays have been described in detail elsewhere [27]. SRB (Alfa-Aeser, Heysham, UK) stock solution was prepared in 1% (*v*/*v*) acetic acid at a concentration of 0.057% (*v*/*v*). A 100 µL volume of cold TCA 10% (*v*/*v*) (Trichloroacetic acid, Sigma-Aldrich, Gillingham, UK) was used to fix HEK 293 cells which were previously treated with thymol solutions and incubated at 4 °C for 1 h. The TCA was removed under a gentle stream of running tap water, then the plate was left to air dry at room temperature. The cells were stained with SRB solution (100 µL/well) for 30 min incubated at room temperature, then SRB was aspirated, and the cells were washed quickly with 1% (*v*/*v*) acetic acid to remove the excess dye. The SRB was extracted with 200 µL of 10 mM Tris base solution (Sigma-Aldrich, Gillingham, UK), having a pH of 10.5. The absorbance was measured at λ_540nm_ and finally, the biocompatibility was calculated as the percentage of the absorbance of treated to untreated cells.

### 4.13. Imaging of HEK293 Cells

HEK293 cells with a density of 2.0 × 10^5^/2 mL were seeded in a 6-well plate and incubated for 24 h. The cells were treated with thymol and Thymol poloxamer at a concentration of 250 and 125 mg/L. Images are acquired for cells stained with NR and SRB using a phase contrast microscope (Nikon Eclipse TS100, Surbiton, UK). 

## 5. Conclusions

Biofilm formation is considered the initial stage of pathogenicity in oral candidiasis. Inhibition of biofilm formation can control the infection and avoid the use of systemic antifungals. Poloxamer 407-Thymol combination showed complete inhibition of the formation of viable Candida biofilm and improved the biocompatibility to a human cell line. This effect is considered a promising effect in the prophylaxis treatment of oral candidiasis.

## Data Availability

The data presented in this study are available in article.
